# Genotoxic and Toxicopathological Responses to Ethylparaben in Plants: Potential Impacts to Crop Yields

**DOI:** 10.3390/toxics13110968

**Published:** 2025-11-10

**Authors:** Edson Araujo de Almeida, Maria Eduarda Nardes Pinto, Ana Elisa Maehashi, Mateus Antônio Vicente Rodrigues, Emily de Moura Galdino, Diego Espirito Santo, Carmem Lúcia Henrich, Osvaldo Valarini Junior, Gideã Taques Tractz, Regiane da Silva Gonzalez, C. A. Downs, Ana Paula Peron

**Affiliations:** 1 Postgraduate Program in Chemistry, Maringá State University, Maringá 87020-900, PR, Brazil; pg55523@uem.br; 2 Environmental Engineering Course, Federal Technological University of Paraná, Campo Mourão 87301-899, PR, Brazil; mariap.1998@alunos.utfpr.edu.br (M.E.N.P.); mateusantonio@alunos.utfpr.edu.br (M.A.V.R.); emilygaldinomoura@alunos.utfpr.edu.br (E.d.M.G.); 3 Chemical Engineering Course, Federal Technological University of Paraná, Campo Mourão 87301-899, PR, Brazil; anamaehashi@alunos.utfpr.edu.br; 4 Postgraduate Program in Biological Sciences, State University of Londrina, Londrina 86020-120, PR, Brazil; diegoespst.1997@uel.br; 5 Postgraduate Program in Environmental Engineering, Federal Technological University of Paraná, Francisco Beltrão 85600-001, PR, Brazil; henrich@alunos.utfpr.edu.br; 6 Postgraduate Program in Food Technology, Federal Technological University of Paraná, Campo Mourão 87301-899, PR, Brazil; osvaldovalarini@utfpr.edu.br (O.V.J.); regiane@utfpr.edu.br (R.d.S.G.); 7 National Network in Management and Regulation of Water Resources, Campo Mourão 87301-899, PR, Brazil; gideatractz@utfpr.edu.br; 8 Haereticus Environmental Laboratory, P.O. Box 85, Gladstone, VA 24553, USA

**Keywords:** paraben, phytotoxicity, cytotoxicity, genotoxicity, aneugenic potential, oxidative stress, agricultural productivity, environment

## Abstract

Ethylparaben (EtP) is an emerging pollutant that is widely found in the environment, particularly in agricultural landscapes. With the extensive contamination of agricultural soils and irrigation waters, there is a rising concern about their potential impact on crop yields. To provide some of the first evidence that EtP may be more than just an agricultural contaminant, but a potential pollutant, we evaluated the systemic toxicities and cellular responses triggered by EtP in seed roots of *Daucus carota*, *Lycopersicum esculentum*, and *Cucumis sativus*, and in bulb roots of *Allium cepa*, at environmentally relevant concentrations of 1, 10, 100, and 1000 ng·L^−1^. The seeds and bulbs remained in contact with the concentrations for 7 days. Distilled water and Tween 80 at 1000 ng·L^−1^ were used as negative controls. The results were subjected to Kruskal–Wallis analysis of variance followed by Dunn’s test (*p* ≤ 0.05). In all plants, all concentrations significantly altered the activity of catalase, ascorbate peroxidase, guaiacol peroxidase, and superoxide dismutase. In carrot (10, 100, and 1000 ng·L^−1^), tomato (1000 ng·L^−1^), and cucumber (all concentrations), such concentrations caused lipid peroxidation, leading to the accumulation of hydrogen peroxide, as well as hydroxyl and superoxide radicals in the cells. These oxidants caused a delay in the progression of the cell cycle and alterations to the mitotic spindle in the root meristems, significantly inhibiting root growth in the plants evaluated. Recurrent contamination with EtP can potentially harm soil quality, posing a risk to both agricultural productivity and the environment.

## 1. Introduction

**Figure 1:**
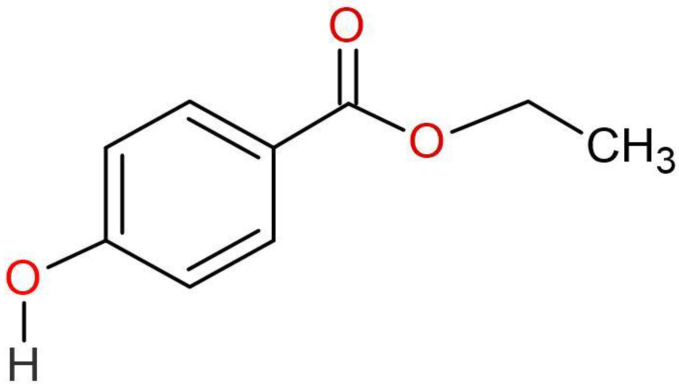
Chemical structure of ethylparaben (own authorship).

Parabens are used to extend the shelf life of processed foods, medicines, cosmetics, and personal care products [1,2], with the industry’s annual utilization exceeding 8000 tons [3,4]. These compounds are short alkyl chain esters of para-hydroxybenzoic acid, and the most commonly used as preservatives are those with open and linear chains, such as methylparaben, ethylparaben, propylparaben, and butylparaben [5]. Among these compounds, ethylparaben (EtP) (Figure 1), is the most widely used industrially because it has a broad antimicrobial spectrum against fungi and bacteria, moderate solubility, good stability at different temperatures and pHs [6], with acute toxicity to humans [5,7,8], as well as being odorless, colorless, and low volatility [7].

However, EtP may contaminate surface water, groundwater, wastewater, drinking water, sludge, and soil [9,10,11,12]. Therefore, this compound is classified as an emerging pollutant, as there is no regulation controlling its release into various environmental matrices, partly because its adverse effects on different ecosystems are still relatively uncharacterized [5,6,13,14]. Furthermore, EtP is classified as a pseudo-persistent compound because its degradation in water and soil is relatively fast (on average, fifteen days). However, its release into the environment is uninterrupted [5].

In water resources, the occurrence of EtP is primarily due to the inefficiency of conventional wastewater treatments in completely removing it from domestic and industrial effluents, resulting in rivers and lakes at concentrations in the ng·L^−1^ range [7,12,15]. Studies with aquatic organisms have shown that EtP can cause neurobehavioral changes, oxidative stress, and bioaccumulation in fish, as well as reduced heart rate, impaired blood circulation, pericardial edema, notochord deformation, and yolk sac abnormalities in fry [8,16]. In addition, EtP has been shown to have the potential to cause cardiac and neurobehavioral changes, immobilization, and death in microcrustaceans, estrogenic effects in copepods, and reduced luminescence in bacteria [17,18,19]. In vertebrate systems, this compound is genotoxic, causing shortening of telomeres, induction of micronuclei, and is associated with an increased risk of cancers, especially estrogen-sensitive cancer lines [20,21,22].

In soil, EtP contamination occurs mainly through the incorporation of domestic sewage sludge into agricultural soils for fertilization purposes, the irrigation of crop areas with contaminated water, and leaching from landfills and planting soils [5,23], being found in these matrices in concentrations ranging from ng to µg·L^−1^ [7,24,25]. Agricultural crop species can absorb parabens and accumulate in their tissues [26,27,28]. Bacterial pathogen contamination of leafy green crops is a serious concern. It often results from either livestock or human waste contamination applied to the crop fields [29,30,31,32]. To mitigate bacterial contamination of leafy greens, one concept (concept only) is to use parabens as part of an integrated pathogen management plan [33,34]. Thus, potential contamination/application from both types of sources requires an assessment of the ecotoxicological impacts of EtP on crops and keystone species of sustainably agricultural landscapes.

*Daucus carota* L. (carrot), *Lycopersicum esculentum* (tomato), and *Cucumis sativus* L. (cucumber) are vegetables widely used in ecotoxicological analyses of organic contaminants, based mainly on physiological and biochemical parameters [35,36,37,38,39]. These species are recommended by the United States Environmental Protection Agency (USEPA) [40] and the Organization for Economic Cooperation and Development (OECD) [41]. The roots of *Allium cepa* L. (onion) bulbs have been used for five decades in ecotoxicological assessments of environmental contaminants worldwide [42,43,44,45], studying physiological, cytological, and biochemical parameters. The results obtained through this test system are similar to those obtained through other biomodels, such as other plants, animals, and cell cultures [39,42,43,44,46,47,48,49].

In this study, the systemic and cellular toxicity of EtP was evaluated at concentrations of 1, 10, 100, and 1000 ng·L^−1^ in *D. carota*, *L. esculentum*, *C. sativus*, and *A. cepa*, based on physiological, cytogenetic, and biochemical markers. This study is expected to enhance the understanding of the ecotoxicity of EtP in soil organisms, as well as promote the development of regulations to control the release of parabens into the environment.

## 2. Materials and Methods

### 2.1. Obtaining EtP and Defining and Preparing Concentrations for Study

The ethylparaben (ethyl para-hydroxybenzoate)—CAS 120-47-8 [50], molecular weight 166.17 g·mol^−1^ and log Kow 2.47—was obtained in analytical grade from Sigma-Aldrich^®^ (St Louis, MO, USA) as were the other reagents used.

Identification and quantification assessments carried out in different countries have shown that EtP has been found in soils at concentrations ranging from 1 ng·L^−1^ to 170 µg·L^−1^ [5,7], and in wastewater and domestic sewage sludge at concentrations from 195.3 to 250 µg·L^−1^ [24,25]. Although the concentrations mentioned are mainly in the µg range, this study evaluated concentrations in ng (1, 10, 100, and 1000 ng·L^−1^) to obtain as close as possible to absolute environmental concentrations, as EtP is pseudo-persistent in water and soil.

EtP is a low-polarity compound and therefore has low solubility in water. To ensure the solubility of this compound in aqueous solution, all experiments were performed in the presence of Tween 80, a low-toxicity solubilizer [38,49,51,52]. Thus, EtP concentrations were prepared in an aqueous medium using Tween 80 at the same mass concentration as the micropollutant. To assess the reliability of EtP toxicity data in the presence of the solubilizer, a negative experimental control was performed using only Tween 80 at a concentration of 1000 ng·L^−1^. In addition, the results obtained were correlated with the positive control, in which a fixed concentration of EtP was used for different concentrations of Tween 80 (1, 10, 100, and 1000 ng·L^−1^). Data are presented in the Supplementary Materials.

### 2.2. EtP Stability Analysis in Aqueous Media

A 0.03 g·L^−1^ EtP stock solution was prepared to assess the stability of this compound in aqueous media for 7 days without light (Equation (1)). The analyses were performed using a UV-Vis spectrophotometer at 255 nm, and the results are presented as percentages.

S (%) = (A_s_/A_0_) × 100 (1)

where S is the stability, A_s_ is the absorbance of the sample, and A_0_ is the initial absorbance on day 0.

### 2.3. Plant Assays

#### 2.3.1. Evaluation of Phytotoxic Potential in Seeds of *D. carota*, *L. esculentum*, and *C. sativus*

The OECD [41] protocol was used to analyze the phytotoxic potential in seeds, and the plants used were *D. carota*, *L. esculentum*, and *C. sativus*. The seeds of these species, free of pesticides and non-transgenic, were purchased from an agricultural supply store. According to the packaging information, the seeds had a germination rate greater than 98% and purity ranging from 99% to 100%. Seeds from the same batch were used for all analyses. Seeds from each plant were distributed in previously sterilized Petri dishes lined with filter paper. Twenty seeds were used per dish, and each concentration was evaluated in quintuplicate. The filter paper was moistened in each dish with up to 1.5 mL of the treatment solution (concentration). The dishes were wrapped in plastic film and placed in a Biochemical Oxygen Demand (BOD) incubator at 25 °C, in the dark, for 7 days. Distilled water and Tween 80 (1000 ng·L^−1^) were used as controls.

The germination percentage was calculated according to Equation (2), and a seed was considered germinated after radicle emergence.

G (%) = (SG/TS) × 100(2)

where G is the germination, SG is the number of seeds germinated, and TS is the total number of seeds used.

After 7 days, the rootlets were measured with a digital caliper and the Relative Growth Index was calculated (Equation (3)).

RGI = RLI/RLC(3)

where RGI is the Relative Growth Index, RLI is the average length of the roots exposed to the treatment, and RLC is the average length of the control roots. According to Biruk et al. [53], an RGI between 0.9 and 1.2 indicates that the treatment does not affect root growth, an RGI below 0.9 indicates inhibition of root elongation, and an RGI greater than 1.2 (RGI > 1.2) indicates stimulation of root growth.

The Germination Index (GI) was calculated according to Equation (4). According to Mañas and Heras [54], a GI equal to or less than 50% characterizes high danger to the plant with acute lethal potential, a GI between 50 and 80% characterizes moderate danger, and a GI greater than or equal to 80% characterizes low danger.

GI (%) = [(RLI × GSI/RLC × GSC)] × 10(4)

where GI is the Germination Index, RLI is the average length of roots exposed to treatment, RLC is the average length of roots in the control, GSI is the number of seeds germinated under exposure to treatment, and GSC is the number of seeds germinated in the control.

#### 2.3.2. Analysis of Phytotoxic, Cytotoxic, and Genotoxic Potential in Roots of *A. cepa* Bulbs

The analyses with *A. cepa* bulb roots were performed according to Fiskesjö [55] with modifications by Nascimento et al. [38] and Filipi et al. [56]. The onion bulbs were purchased from an organic garden. Before starting the experiments, the cataphylls and dry roots were removed from the onions and washed in distilled running water. The clean onions were placed in bottles with the treatment solutions (concentrations) in which the root growth zone was submerged, and then placed in a BOD incubator for 7 days without light. The treatment solutions were prepared and changed daily. Each concentration was evaluated in quintuplicate, and distilled water and Tween 80 (1000 ng·L^−1^) were used as controls.

After 7 days of incubation in the BOD, the lengths of five roots from each onion were measured using a digital caliper. The Average Root Length (ARL) was calculated for each concentration according to Equation (5) to determine the phytotoxic potential.

ARL (cm) = Sum of root length/5(5)

Additionally, roots were collected from each bulb and fixed in Carnoy’s solution (3:1) for 24 h. Slides were then prepared from the meristematic regions of these roots, which were analyzed under an optical microscope at 40× magnification.

To evaluate the cytotoxic potential, 2000 cells from each onion were analyzed, totaling 10,000 cells per concentration, and the Mitotic Index (MI) was calculated (Equation (6)). To evaluate genotoxicity, 200 cells from each onion were analyzed, totaling 2000 cells analyzed per concentration, and the Cellular Alteration Index (CAI) was calculated (Equation (7)). The cellular alterations considered in the analyses were: polyploidy, micronuclei, chromosomal abnormalities in different phases of mitosis, and chromosomal breakage.

MI = (DC/TC) × 100(6)

CAI = (AC/TC) × 100(7)

where MI is the Mitotic Index, DC is the dividing cells, TC is the total number of dividing cells, CAI is the Cell Alteration Index, and AC is the altered cells.

### 2.4. Enzymatic Analysis in Seed and Bulb Roots

#### 2.4.1. Preparation of Roots for Enzymatic Analysis

Fifty mg of seed root and bulb roots meristems from each replicate were macerated in 1 mL of HCl (38%) and 2 mL of diethylenetriaminepentaacetic acid (5 mM), and then centrifuged at 4000 rpm for 15 min to obtain the enzyme extracts.

The enzyme extracts obtained were analyzed in a UV-Vis spectrophotometer to evaluate the modulation of catalase (CAT), ascorbate peroxidase (APX), guaiacol peroxidase (GPOX), and superoxide dismutase (SOD) enzymes.

#### 2.4.2. Enzymatic Analysis

CAT activity was analyzed based on the method described by Kraus et al. [57], with adaptations proposed by Azevedo et al. [58]. 2.5 mL of sodium phosphate buffer (pH 7.8) was added to 100 µL of the enzyme extract for each sample. Then, 1 mL of 1 mM hydrogen peroxide (H_2_O_2_) was added to read the enzyme activity at 240 nm. The extinction coefficient used for the calculations was 2.8 M^−1^·cm^−1^, and the results were expressed in µmol·min^−1^·µg^−1^ of protein (Equation (8)).

U = {[(A/t)/E] × Ve × DF}/P(8)

where U is the enzyme unit, A is the measured absorbance, t is the analysis time, E is the extinction coefficient, Ve is the enzyme volume, DF is the dilution factor, and P is the protein, obtained from the mass of roots used.

APX activity analysis was performed according to the method by Zhu et al. [59]. To the enzyme extracts, 2.5 mL of sodium phosphate buffer, 500 µL of 0.25 mM ascorbic acid, and 1 mL of 1 mM hydrogen peroxide (H_2_O_2_) were added. The reading was performed at 290 nm. An extinction coefficient of 2.8 M^−1^·cm^−1^ was used, and the results were expressed in µmol·min^−1^·µg^−1^ of protein (Equation (8)).

The analysis of GPOX activity was performed based on the protocol by Matsuno and Uritani [60]. To 300 µL of the enzyme extract, 2.5 mL of sodium phosphate buffer, 250 µL of 0.1 M citric acid, and 250 µL of 0.5% guaiacol were added. Next, 250 µL of 1 mM hydrogen peroxide (H_2_O_2_) was added. The mixtures were homogenized in a vortex and incubated in an oven at 30 °C for 15 min. After this period, the samples were cooled in an ice bath for 10 min, and 250 µL of 2% sodium metabisulfite was added. The reading was performed at 450 nm. The extinction coefficient used was 26.6 M^−1^·cm^−1^, and the results were expressed in µmol·min^−1^·µg^−1^ of protein (Equation (8)).

The SOD assay was performed according to the protocol described by Sun et al. [61]. Samples were prepared in duplicate: half of the extract aliquots were exposed to 80 W fluorescent light for 20 min, while the other half were kept in the dark. To 200 µL of each aliquot, 0.8 mL of sodium phosphate buffer, 500 µL of 0.1 mM ethylenediaminetetraacetic acid (EDTA), 500 µL of methionine, 500 µL of nitro blue tetrazolium (NBT), and 200 µL of riboflavin were added. Absorbance was measured at 560 nm, and SOD activity was expressed as U per protein (Equation (9)).

SOD = {[(B_l_ − S_l_)]/B_l_] − [(B_e_ − S_e_)]/B_e_]}/50 (9)

where B_l_ is the absorbance of the blank kept in the light prepared without the enzyme extract, S_l_ is the absorbance of the sample kept in the light, B_e_ is the absorbance of the blank kept in the dark, and se is the absorbance of the sample kept in the dark. The quotient 50 represents the amount of enzyme required to inhibit 50% of the photoreduction of NBT.

### 2.5. Analysis of Phenolic Content (Folin–Ciocalteu) and Lipid Peroxidation (TBARs)

#### 2.5.1. Sample Preparation

Fifty mg of seed root and bulb roots meristems from each replicate were centrifuged in distilled water at 4000 rpm for 15 min to obtain homogenized supernatants.

#### 2.5.2. Folin–Ciocalteu (FC) Assay

The Folin–Ciocalteu (FC) assay was performed following the protocol of Carmona-Hernandez et al. [62]. To 50 µL of homogenate, 100 µL of FC reagent (0.0288 g of phosphotungstic acid and 0.0182 g of phosphomolybdic acid dissolved in 5 mL of methanol), 50 µL of ethanol, and 50 µL of distilled water were added. The mixtures were kept in the dark for 10 min, after which 50 µL of saturated sodium bicarbonate solution was added, and the samples were incubated in darkness for an additional 50 min. After incubation, the samples were centrifuged at 4000 rpm for 5 min, and the supernatants were analyzed. Absorbance was measured spectrophotometrically at 745 nm.

#### 2.5.3. TBARs Assay on Roots

Lipid peroxidation was assessed following the method of Papastergiadis et al. [63]. To 50 µL of homogenate, 250 µL of TBARs solution (46 mM) was added, and the mixture was incubated in a water bath at 90 °C for 35 min. After cooling, absorbance was measured spectrophotometrically at 532 nm.

### 2.6. Statistical Analysis

The results of phytotoxicity, cytotoxicity, genotoxicity, antioxidant enzyme activity, and non-enzymatic biochemical tests were submitted to Kruskal–Wallis analysis of variance followed by Dunn’s test (*p* ≤ 0.05), as the data were deemed non-normal by Lilliefors’ test.

## 3. Results

### 3.1. Effect of EtP on Solubilizer Independence

Given the need to solubilize EtP in water using Tween 80, an experimental model was developed to demonstrate the effect of EtP independently of the solubilizer. The results, presented as Supplementary Materials (Figures S1–S5), demonstrated that the solubilizer did not interfere with the data obtained for the micropollutant, evidencing the non-toxicity of Tween 80 and the dose-dependent effect of EtP on the experimental models used.

### 3.2. Stability in Aqueous Media

**Figure 2:**
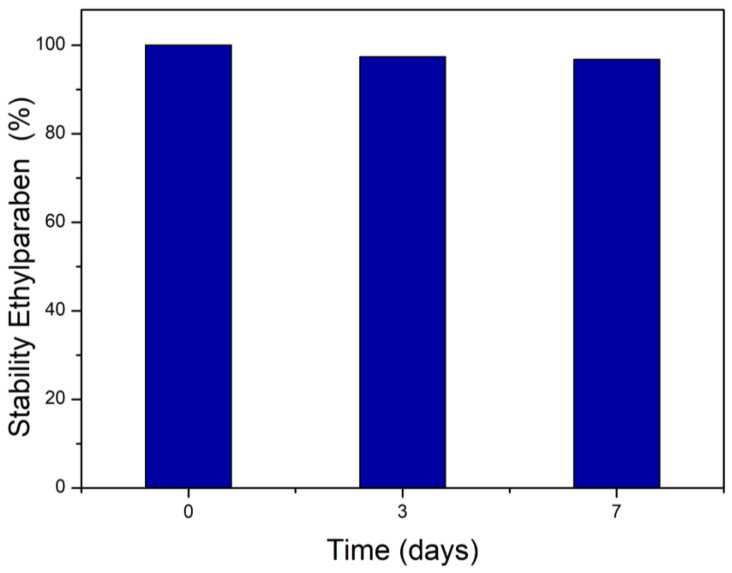
Stability of ethylparaben in aqueous media for 7 days in the absence of light.

In order to provide rigor to the experimental design, the stability of EtP in aqueous media was evaluated at a concentration of 0.03 g·L^−1^ (Figure 2). It was observed that there was no change in the concentration over 7 days, demonstrating that the compound remained stable in aqueous media.

### 3.3. Toxicity in Seeds of D. carota, L. lycopersicim, and C. sativus and in Roots of A. cepa

**Figure 3:**
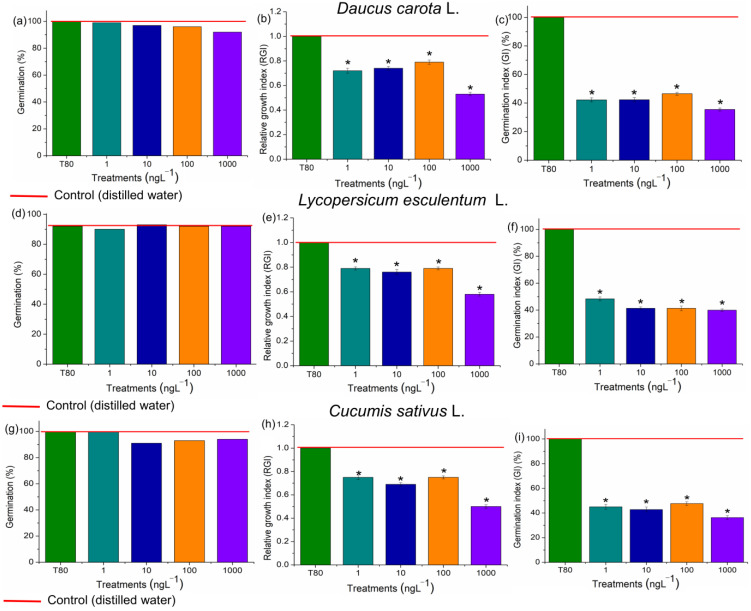
Phytotoxic potential of ethylparaben on seeds of *Daucus carota* L., *Lycopersicum sculentum* L., and *Cucumis sativus* L., at concentrations of 1, 10, 100, and 1000 ng·L^−1^, based on the parameters Seed Germination (**a**,**d**,**g**), Relative Growth Index (**b**,**e**,**h**), and Germination Index (**c**,**f**,**i**). * Significant difference between the controls, according to Kruskal–Wallis H, followed by Dunn’s post hoc test (*p* ≤ 0.05). T80—Tween 80 (1000 ng·L^−1^).

The four concentrations of EtP evaluated did not harm seed germination in *D. carota*, *L. esculentum*, and *C. sativus* (Figure 3a,d,g). However, they significantly reduced root growth in these plants, which showed a Relative Growth Index (RGI) of 0.9 or less (Figure 3b,e,h). The Germination Index (GI) is the ability of the seed to germinate, elongate rootlets, and form a healthy plant [54]. However, the GI obtained in this study for the three vegetables was less than 50% (Figure 3c,f,i), indicating that EtP is harmful and has the potential for acute lethality.

**Figure 4:**
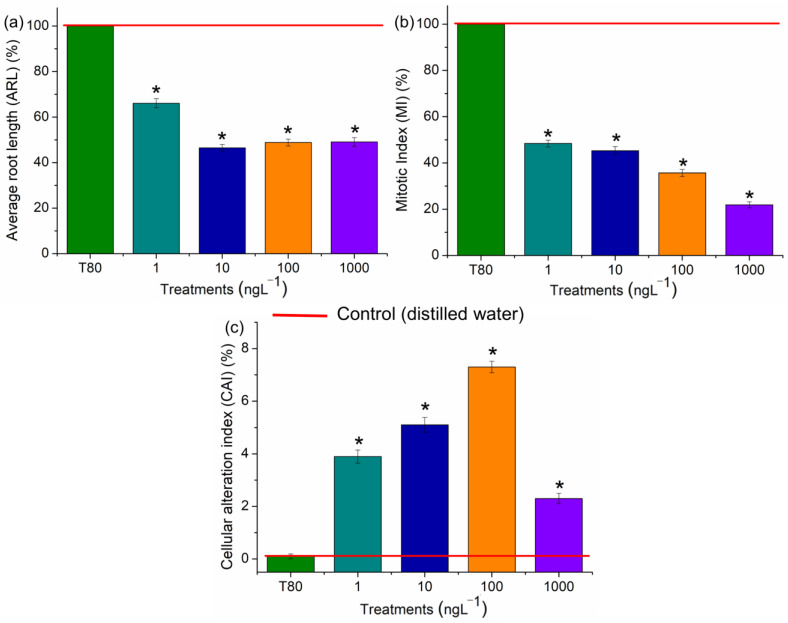
Phytotoxic, cytotoxic, and genotoxic potential of ethylparaben, at concentrations of 1, 10, 100, and 1000 ng·L^−1^, in *Allium cepa* L. bulb roots, based on the parameters Average Root Length (ARL) (**a**), Mitotic Index (MI) (**b**) and Cell Alteration Index (CAI) (**c**). * Significant difference between the controls, according to Kruskal–Wallis H, followed by Dunn’s post hoc test (*p* ≤ 0.05). T80—Tween 80 (1000 ng·L^−1^).

**Figure 5:**
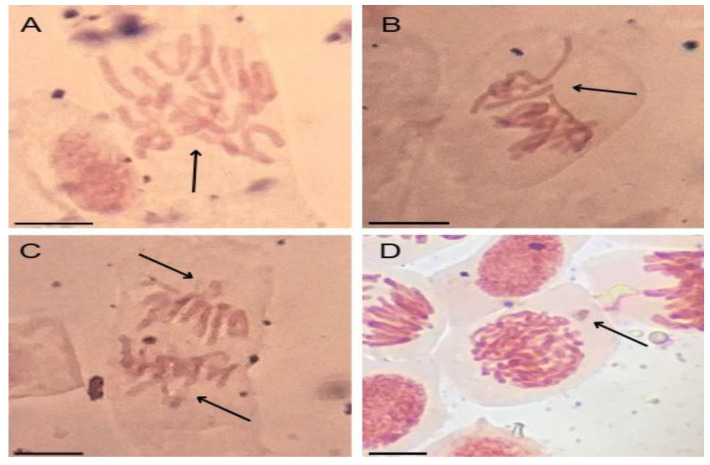
Cellular alterations observed in root meristems of *Allium cepa* L. bulbs exposed to ethylparaben at concentrations of 1, 10, 100, and 1000 ng·L^−1^. (**A**) chromosome disorder in metaphase, (**B**) chromosome disorder in metaphase with chromosome loss, (**C**) chromosome loss in anaphase, and (**D**) micronuclei. Bar: 10 µm.

All concentrations of EtP were phytotoxic to *A. cepa* bulbs, causing a significant reduction in root growth (Figure 4a), which corroborates the results of inhibition of root elongation in *D. carota*, *L. esculentum*, and *C. sativus* (Figure 3b,e,h). Furthermore, EtP at all concentrations caused a significant mitodepressive effect on onion root meristems, resulting in cell division rates of less than 50% compared to the controls (Figure 4b). All concentrations induced cellular changes at a significant frequency (Figure 4c), showing that they are cytotoxic to the bulbs’ roots. Furthermore, chromosomal toxicities in roots of *A. cepa* were observed specifically regarding chromosome disorganization in metaphase (Figure 5A), chromosome disorganization in metaphase with chromosome loss (Figure 5B), chromosome loss in anaphase (Figure 5C), and the formation of micronuclei (Figure 5D).

### 3.4. Oxidative Stress in Roots of D. carota, L. esculentum, and C. sativus, and in Roots of Bulbs of A. cepa

**Figure 6:**
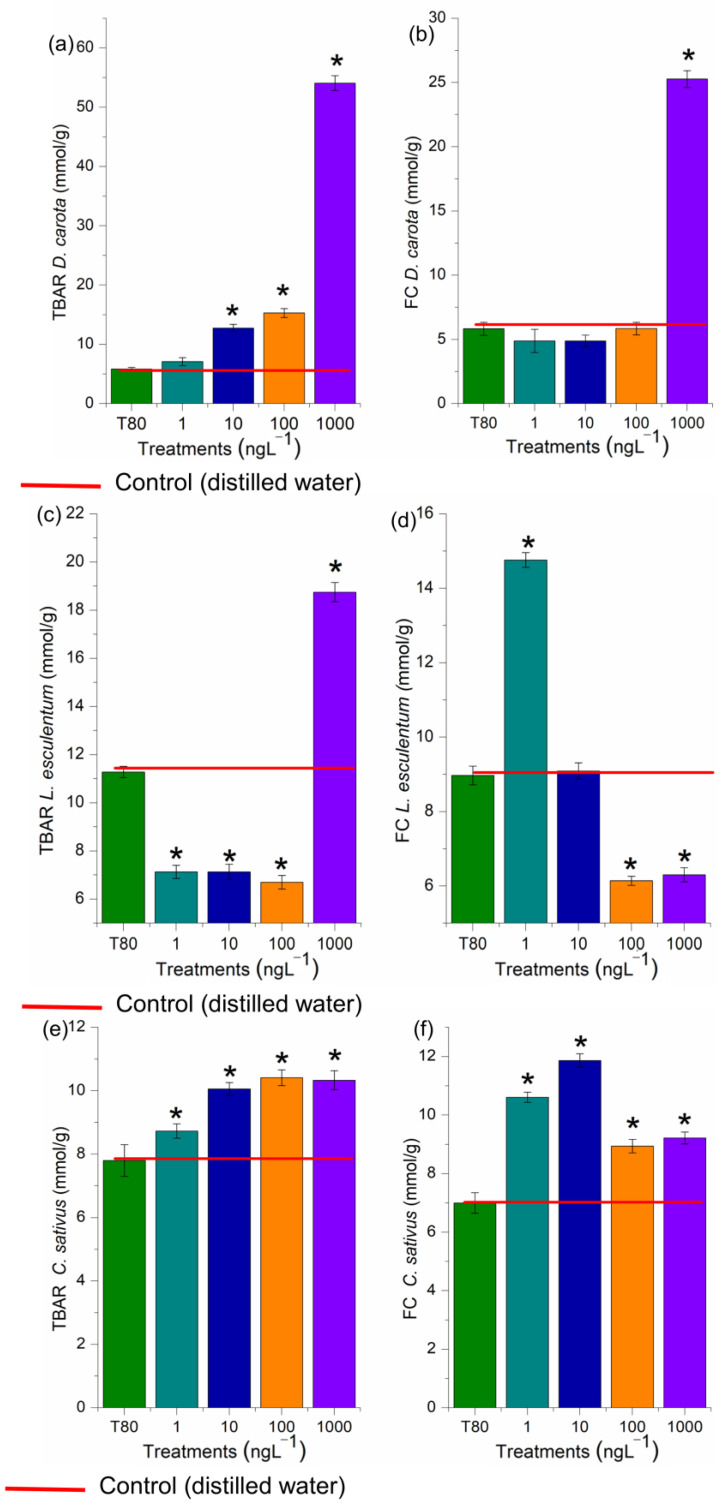
Lipid peroxidation (TBARs) (**a**,**c**,**e**) and concentration of phenolic compounds (FC) (**b**,**d**,**f**) in rootlets of *Daucus carota* L., *Lycopersicum esculentum* L., and *Cucumis sativus* L. exposed to ethylparaben at concentrations of 1, 10, 100, and 1000 ng·L^−1^. * Significant difference from the controls was observed using a Kruskal–Wallis H, followed by Dunn’s post hoc test (*p* ≤ 0.05). T80—Tween 80 (1000 ng·L^−1^).

**Figure 7:**
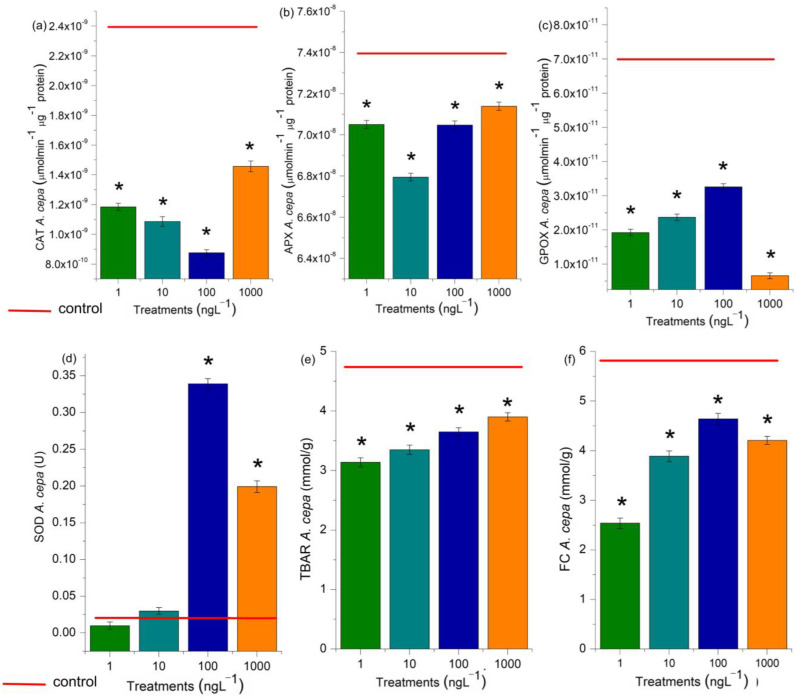
Modulations of the enzymes catalase (CAT) (**a**), ascorbate peroxidase (APX) (**b**), guaiacol peroxidase (GPOX) (**c**), and superoxide dismutase (SOD) (**d**), and lipid peroxidation (TBARs) (**e**) and concentration of phenolic compounds (FC) (**f**), in *Allium cepa* L. bulb roots exposed to ethylparaben in concentrations 1, 10, 100, e 1000 ng·L^−1^. * Significant difference between the controls according to Kruskal–Wallis H, followed by Dunn’s post hoc test (*p* ≤ 0.05). T80—Tween 80 (1000 ng·L^−1^).

Enzymatic and non-enzymatic biochemical markers are essential for assessing the harmfulness and understanding how systemic and cellular toxicity is triggered [38]. In this study, the concentration 1000 ng·L^−1^ in *D. carota* (Figure 6b), 1 ng·L^−1^ in *L. esculentum* (Figure 6d), and 1, 10, 100, and 1000 ng·L^−1^ in *C. sativus* (Figure 6f), overactivated the root defense system since it significantly increased the concentration of phenolic compounds in the meristems, demonstrating that EtP caused an imbalance in cell function. However, EtP at 100 and 1000 ng·L^−1^ in *L. esculentum* (Figure 6d) and at 1, 10, 100, and 1000 ng·L^−1^ in *A. cepa* bulbs (Figure 7f) caused a significant reduction in the concentration of these compounds, making the cells vulnerable to stressors.

**Figure 8:**
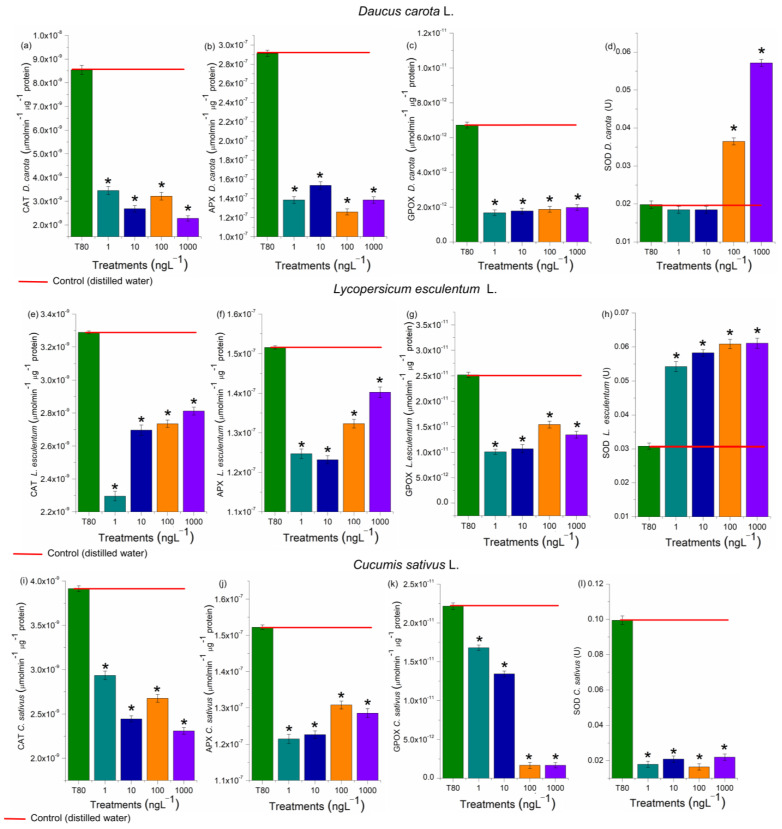
Modulations of the enzymes catalase (CAT) (**a**,**e**,**i**), ascorbate peroxidase (APX) (**b**,**f**,**j**), guaiacol peroxidase (GPOX) (**c**,**g**,**k**), and superoxide dismutase (SOD) (**d**,**h**,**l**) in roots of *Daucus carota* L., *Lycopersicum esculentum* L., and *Cucumis sativus* L. exposed to ethylparaben in concentrations 1, 10, 100, e 1000 ng·L^−1^. * Significant difference between the controls according to Kruskal–Wallis H, followed by Dunn’s post hoc test (*p* ≤ 0.05). T80—Tween 80 (1000 ng·L^−1^).

The activity of CAT, APX, SOD, and GPOX was evaluated in the radicles of *D. carota*, *L. esculentum*, and *C. sativus*, and the roots of *A. cepa* bulbs (Figure 7 and Figure 8). These enzymes are responsible for maintaining the cell cycle’s homeostasis, which is essential for cell proliferation in meristems and plays a crucial role in plant growth and development [38]. At all EtP concentrations and in all plant species, there was a significant reduction in the activity of CAT, APX, and GPOX (Figure 7a–c and Figure 8a–c,e–g,i–k), potentially resulting in the accumulation of hydrogen peroxide and hydroxyl radicals in the cells. Furthermore, the 100 and 1000 ng·L^−1^ concentrations in bulbs and carrots (Figure 7d and Figure 8d), as well as all concentrations in tomatoes (Figure 8h), significantly increased SOD activity, demonstrating that EtP induced the production of superoxide radicals in tissues. In cucumber, SOD was expressly inhibited (Figure 8l), exposing the cells to these radicals.

Based on the TBARs test (Figure 6), the concentrations of 10, 100, and 1000 ng·L^−1^ in *D. carota* (Figure 6a), 1000 ng·L^−1^ in *L. esculentum* (Figure 6c), and all four concentrations in *C. sativus* (Figure 6e) caused lipid peroxidation in the roots. The compounds resulting from lipid oxidation in cell membranes are hydroxyl radicals, hydroperoxyl radicals, ketones, aldehydes, and carboxylic acids, which are highly reactive to cell proteins [64]. Thus, the reduction in CAT, APX, and GPOX activity (Figure 7a–c and Figure 8a–c,e–g,i–k), as well as the lipid peroxidation observed in carrots and cucumbers (Figure 6a,e), corroborate the high concentration of phytochemicals produced in the roots of these plants (Figure 6b,d,f), which tried to contain the production of oxidizing radicals in the cells in order to preserve root growth, but without success (Figure 3 and Figure 4).

In this study, EtP at 1000 ng·L^−1^ in *D. carota* (Figure 6b), at 1 ng·L^−1^ in *L. esculentum* (Figure 6d), and at 1, 10, 100, and 1000 ng·L^−1^ in *C. sativus* (Figure 6f), overactivated the root defense system since it significantly increased the concentration of phenolic compounds in the meristems, demonstrating that this antimicrobial caused an imbalance in cell function. However, EtP at 100 and 1000 ng·L^−1^ in *L. esculentum* (Figure 6d) and at 1, 10, 100, and 1000 ng·L^−1^ in *A. cepa* bulbs (Figure 7e) caused a significant reduction in the concentration of these compounds, making the cells vulnerable to stressors.

## 4. Discussion

### 4.1. Histological and Chromosomal Toxicity

Although xenobiotics probably do not cause harm to seed germination, as observed for *D. carota*, *L. esculentum*, and *C. sativus* in the present study (Figure 3), they can be phytotoxic to the development of rootlets since the apoplastic barriers in these structures are not selective to the entry of substances that are anomalous to the metabolism of seedlings [65]. The reduction in root growth, as observed for these three vegetables (Figure 3), characterizes a profound sub-lethal effect on plants, as it reduces the absorption of water and nutrients and can substantially compromise seedling development [66].

The reduction in cell division observed in the root tips of *A. cepa* (Figure 4b) resulted from EtP inducing cell-cycle arrest, likely by disrupting cellular checkpoints in interphase, either through damage to DNA synthesis and/or protein synthesis and/or the DNA repair machinery. EtP caused mitotic indices of less than 50% in meristematic tissues, as observed for concentrations of 1, 10, and 100 ng·L^−1^ (Figure 4b). Cell division rates in meristematic cells of less than 22%—as observed for a concentration of 1000 ng·L^−1^ (Figure 4b)—have the potential to cause acute death/crop failure [67,68].

Chromosomal disorders in metaphases, such as those observed here in *A. cepa* meristems (Figure 5), are considered serious errors in the functioning of meristems since they are caused by the complete inactivation of the mitotic spindle and lead to the asymmetrical segregation of genetic material during cell division, giving rise to aneuploid and polyploid cells after successive divisions [46,69,70]. Cells with chromosomal disorders (Figure 5), which result from the loss or gain of genetic material, exhibit altered metabolism. However, they can be selectively eliminated from young tissues when their functioning is nonviable, further accentuating the inhibition of cell division in meristems [68,71].

Micronuclei are chromosome fragments created from acentric or lagging chromosomes due to deficiencies in the shortening of microtubules during cell division that fail to incorporate into the nucleus during mitosis, such as those observed in this study in metaphase and anaphase (Figure 5B,C) [68,72]. The recurring presence of micronuclei in meristems characterizes high genetic instability. It can manifest as genotypic/phenotypic disturbances to the functioning of plant organs [55,68,73]. Therefore, when considering the ability to cause disturbances to the mitotic spindle (Figure 5), it is inferred that EtP, as well as being genotoxic to root meristems, proved to be a xenobiotic with a high aneugenic potential for roots.

Thus, establishing that EtP causes a significant accumulation of micronuclei in young tissues suggests that this micropollutant, at environmentally relevant concentrations, threatens crop productivity and sustainability. Furthermore, the accumulation of micronuclei in reproductive tissue can result in asymmetrical gametes during meiosis, leading to aborted pollen/egg cells, or the developmental pathology of tapetal cells, which manifests as reduced fertility/viable seed production—a toxic Mechanism of Action [74,75,76]. A second Mechanism of Action between EtP and micronuclei in the reduction in crop productivity is the impairment of growth via inhibition of cell division in meristematic tissue [71,77,78,79,80]. A third Mechanism of Action is the phenotypic consequence of this genotoxicity, which is an increased vulnerability to other environmental stressors, such as heat stress, water stress, and infectious diseases [73,81,82,83]. Therefore, the presence of EtP in amended soils and irrigation water should be avoided during the early phases of crop development. Furthermore, EtP should be used for antimicrobial treatment for human pathogens in green leafy crops only near the end of their growing period, such as for vertical farming of lettuces or post-harvest [33,34].

### 4.2. Oxidative Damage and Oxidative-Stress Homeostatic Responses

Hydrogen peroxide, hydroxyl radicals, superoxide radicals, and radicals from lipid peroxidation cause significant disturbances to the cell cycle in meristems by denaturing proteins/enzymes such as those responsible for DNA duplication, protein synthesis, polymerization/functioning of the mitotic spindle, and chromosome organization in the different phases of mitosis [38,39,49,84,85,86,87]. EtP, at all the concentrations evaluated, induced the formation of significant levels of reactive oxygen species in cells (Figure 6 and Figure 7), which may have contributed cell cycle arrest and mitotic spindle changes in root meristems (Figure 4b,c), significantly inhibiting the growth of rootlets in carrot, tomato, and cucumber (Figure 3b,e h) and of roots in bulbs in A. cepa (Figure 4a). Parabens are documented to induce cell cycle arrest via non-oxidative damage mechanisms; therefore, the relative contribution of oxidative damage and non-oxidative damage processes to the cumulative manifestation of cell cycle arrest needs to be further explored in plants [14,39,88,89,90].

Systemic and cellular toxicity triggered by oxidative stress due to the action of xenobiotics—such as those caused by EtP on plants (Figure 3, Figure 4 and Figure 5)—are among the leading causes of loss of productivity in different crops around the world, since oxidizing compounds cause disturbances to cellular and physiological mechanisms, which can ultimately alter the economics of organismal homeostasis, leading to reduced fitness and crop yields [91,92,93]. In addition to impacting crop yield, the environmental persistence of EtP-contaminated soils poses a threat to subsequent crop plantings, altering the calculations for successful future returns from that contaminated field [94,95,96,97,98].

Todorovac et al. [91] observed that EtP caused a reduction in cell proliferation and induced cellular changes in plants in a 24 h exposure test, corroborating the cytotoxicity and genotoxicity results obtained in the present study. However, the concentrations evaluated by these researchers were in the mg·L^−1^ range, which is very high compared to the actual concentrations of this compound in the environment, which generally range from µg to ng. Nevertheless, the results obtained by these researchers highlight the toxicity results obtained for EtP in the present study, since this compound was evaluated at concentrations thousands of times lower. Phytotoxicity, cytotoxicity, and genotoxicity were observed in the roots at concentrations as low as 1 ng·L^−1^ (Figure 3, Figure 4 and Figure 5). Furthermore, Kim et al. [14] evaluated plants in soils contaminated with EtP at concentrations above 200 mg/kg. They observed a severe reduction in stem and leaf growth and development, as well as the death of organisms. However, they did not report adverse effects on root development/functioning. Thus, the results obtained by Kim et al. [14] and Todorovac et al. [91] complement the results presented here, suggesting that EtP, being toxic to roots, caused a reduction in water and nutrient absorption, which triggered severe changes in the development of the aerial parts of the plants. Furthermore, it should be noted that after a thorough search of the scientific literature, only the two studies mentioned above on evaluating EtP on plants were found, which did not consider the oxidative stress triggered by this antimicrobial in these organisms.

## 5. Conclusions

EtP triggered oxidative stress, which inhibited the progression of the cell cycle and disrupted the organization of chromosomes in metaphase and anaphase, resulting in a significant reduction in root growth across different plants.

The data show the high potential for EtP to not only be an ecological hazard but also a potential threat to both crop productivity and seed viability, reinforcing the need to manage its contamination of agricultural landscapes, both as a component of a soil amendment and an irrigant, as well as its use in hydroponics. If used as a human pathogen mitigation measure in leafy green crops that are eaten raw, applying EtP in the field is not recommended. However, it may be a consideration for post-harvest treatment.

## Data Availability

The original contributions presented in this study are included in the article/Supplementary Materials. Further inquiries can be directed to the corresponding authors.

## Supplementary Materials

The following supporting information can be downloaded at: https://www.mdpi.com/article/10.3390/toxics13110968/s1. Figure S1. Phytotoxic potential of tween 80 (1000 ng·L^−1^) on seeds of *Daucus carota* L., *Lycopersicum sculentum* L., and *Cucumis sativus* L., at concentrations of 1, 10, 100, and 1000 ng·L^−1^, based on the parameters Seed Germination, Relative Growth Index, and Germination Index; Figure S2. Phytotoxic, cytotoxic, and genotoxic potential of and tween 80, at concentrations of 1, 10, 100, and 1000 ng·L^−1^ in *Allium cepa* L. bulb roots, based on the parameters Average Root Length (ARL), Mitotic Index (MI) and Cell Alteration Index (CAI); Figure S3. Lipid peroxidation (TBARs) and concentration of phenolic compounds (FC) in rootlets of *Daucus carota* L., *Lycopersicum esculentum* L., and *Cucumis sativus* L. exposed to tween 80 at concentrations of 1, 10, 100, and 1000 ng·L^−1^; Figure S4. Modulations of the enzymes catalase (CAT), ascorbate peroxidase (APX), guaiacol peroxidase (GPOX), and superoxide dismutase (SOD) in roots of *Daucus carota* L., *Lycopersicum esculentum* L., and *Cucumis sativus* L. exposed to tween 80 in concentrations 1, 10, 100, e 1000 ng·L^−1^; Figure S5. Modulations of the enzymes catalase (CAT), ascorbate peroxidase (APX), guaiacol peroxidase (GPOX), and superoxide dismutase (SOD) in roots of *Daucus carota* L., *Lycopersicum esculentum* L., and *Cucumis sativus* L. exposed to tween 80 in concentrations 1, 10, 100, e 1000 ng·L^−1^.

## Abbreviations

The following abbreviations are used in this manuscript:

EtP	Ethylparaben
BOD	Biochemical Oxygen Demand
RGI	Relative Growth Index
GI	Germination Index
ARL	Average Root Length
MI	Mitotic Index
CAI	Cellular Alteration Index
CAT	Catalase
APX	Ascorbate peroxidase
GPX	Guaiacol peroxidase
SOD	Superoxide dismutase
U	Enzyme unit
FC	Folin–Ciocalteu
