# Supplementary material for: Genotoxic and Toxicopathological Responses to Ethylparaben in Plants: Potential Impacts to Crop Yields
Source: Toxics. 2025 Nov 10;13(11):968. doi: 10.3390/toxics13110968 (PMC12656058; doi:10.3390/toxics13110968)
Supplement: Supplementary file 1 [file toxics-13-00968-s001.zip › toxics-3953401-supplementary.pdf]

toxics

MDPI

Article

Genotoxic and Toxicopathological Responses to Ethylparaben in Plants: Potential Impacts to Crop Yields

Edson Araujo de Almeida [1], Maria Eduarda Nardes Pinto [2], Ana Elisa Maehashi [3], Mateus Antônio Vicente Rodrigues [2], Emily de Moura Galdino [2], Diego Espirito Santo [4], Carmem Lúcia Henrich [5], Osvaldo Valarini Junior [6], Gideã Taques Tractz [7], Regiane da Silva Gonzalez [6], C. A. Downs [8,*] and Ana Paula Peron [5,6,*]

[1] Postgraduate Program in Chemistry, Maringá State University, Maringá 87020-900, PR, Brazil; pg55523@uem.br
[2] Environmental Engineering Course, Federal Technological University of Paraná, Campo Mourão 87301-899, PR, Brazil; mariap.1998@alunos.utfpr.edu.br (M.E.N.P.); mateusantonio@alunos.utfpr.edu.br (M.A.V.R.); emilygaldinomoura@alunos.utfpr.edu.br (E.d.M.G.)
[3] Chemical Engineering Course, Federal Technological University of Paraná, Campo Mourão 87301-899, PR, Brazil; anamaehashi@alunos.utfpr.edu.br
[4] Postgraduate Program in Biological Sciences, State University of Londrina, Londrina 86020-120, PR, Brazil; diegoespst.1997@uel.br
[5] Postgraduate Program in Environmental Engineering, Federal Technological University of Paraná, Francisco Beltrão 85600-001, PR, Brazil; henrich@alunos.utfpr.edu.br
[6] Postgraduate Program in Food Technology, Federal Technological University of Paraná, Campo Mourão 87301-899, PR, Brazil; osvaldovalarini@utfpr.edu.br (O.V.J.); regiane@utfpr.edu.br (R.d.S.G.)
[7] National Network in Management and Regulation of Water Resources, Campo Mourão 87301-899, PR, Brazil; gideatractz@utfpr.edu.br;
[8] Haereticus Environmental Laboratory, P.O. Box 85, Gladstone, Virginia 24553, USA
* Correspondence: cadowns@haereticus-lab.org (C.A.D.); anaperon@utfpr.edu.br (A.P.P.)

Academic Editor: Rafael Clemente

Received: 12 October 2025
Revised: 2 November 2025
Accepted: 7 November 2025
Published: date

Citation: Almeida, E.A.d.; Pinto, M.E.N.; Maehashi, A.E.; Rodrigues, M.A.V.; Galdino, E.d.M.; Santo, D.E.; Henrich, C.L.; Junior, O.V.; Tractz, G.T.; Gonzalez, R.d.S.; et al. Genotoxic and Toxicopathological Responses to Ethylparaben in Plants: Potential Impacts to Crop Yields. *Toxics* **2025**, *13*, x. https://doi.org/10.3390/xxxxx

Copyright: © 2025 by the authors. Submitted for possible open access publication under the terms and conditions of the Creative Commons Attribution (CC BY) license (https://creativecommons.org/licenses/by/4.0/).

Supplementary Information

Figures S1, S2, S3, S4, and S5 show the data from the tests performed with Tween 80 solutions at 1, 10, 100, and 1000 ng.L^{-1} concentrations. It can be observed that for all solubilizer concentrations, the results of the analyses were close to the negative control (distilled water). On the other hand, solutions with EtP (positive control) caused changes in cell proliferation, cell alterations, and oxidative stress regardless of the concentration of Tween 80 used. Thus, the surfactant did not cause toxicity to the plant bioassays used, nor did it interfere with the adverse action of EtP on the test systems.

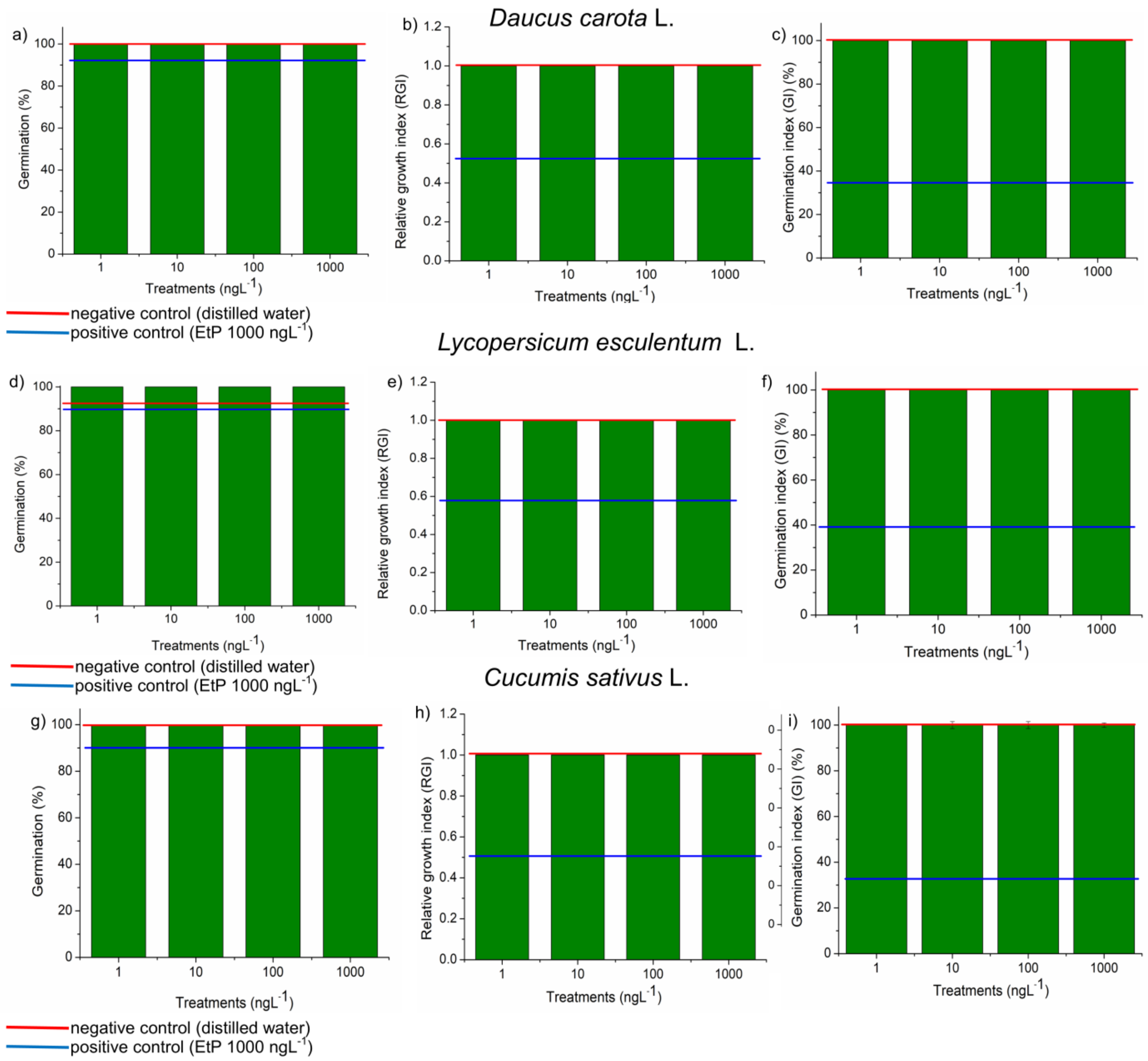

Figure S1. Phytotoxic potential of tween 80 (1000 ng. L^{-1}) on seeds of *Daucus carota* L., *Lycopersicum sculentum* L., and *Cucumis sativus* L., at concentrations of 1, 10, 100, and 1000 ng. L^{-1}, based on the parameters Seed Germination, Relative Growth Index, and Germination Index.

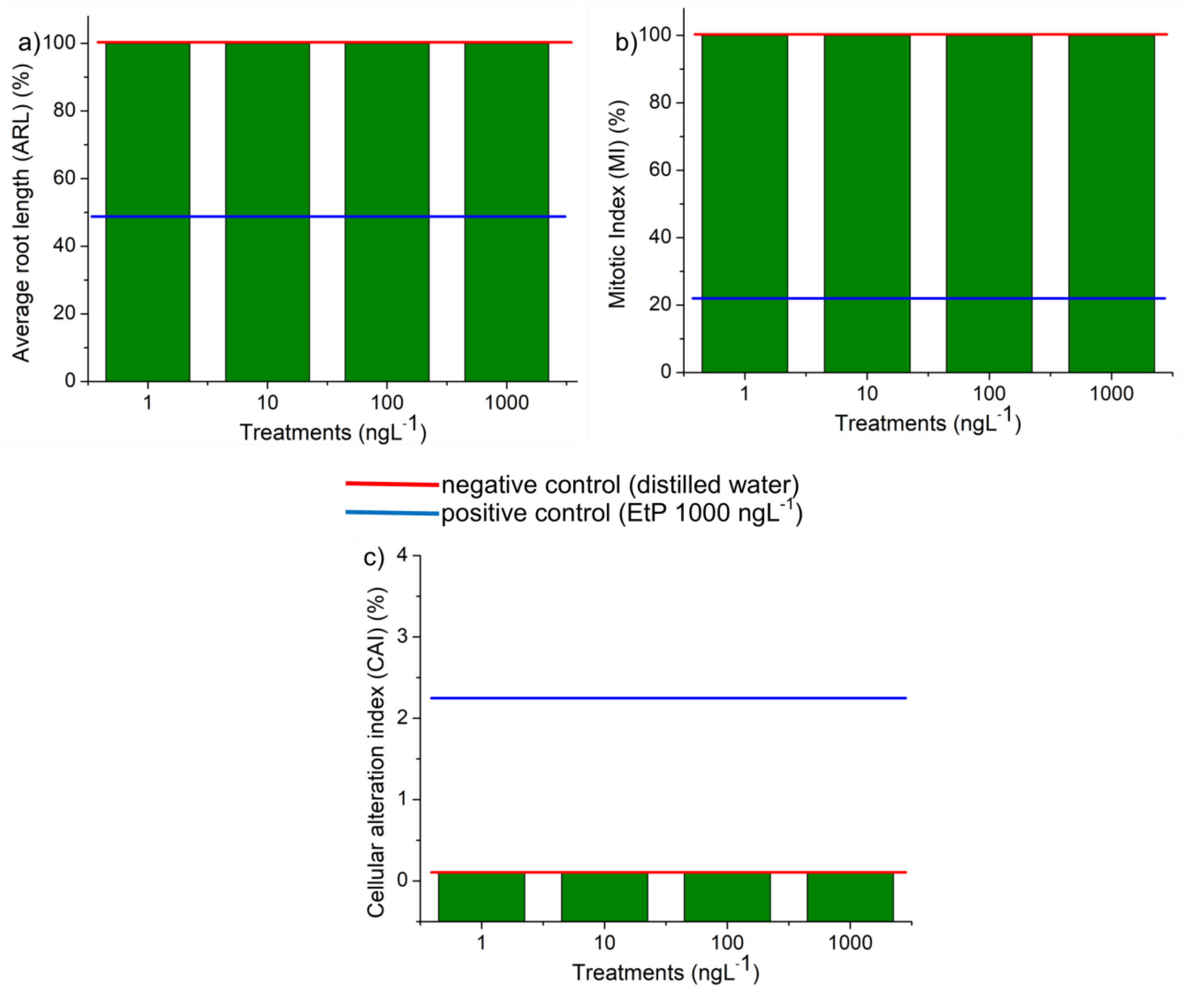

Figure S2. Phytotoxic, cytotoxic, and genotoxic potential of and tween 80, at concentrations of 1, 10, 100, and 1000 ng.L^{-1} in *Allium cepa* L. bulb roots, based on the parameters Average Root Length (ARL), Mitotic Index (MI) and Cell Alteration Index (CAI).

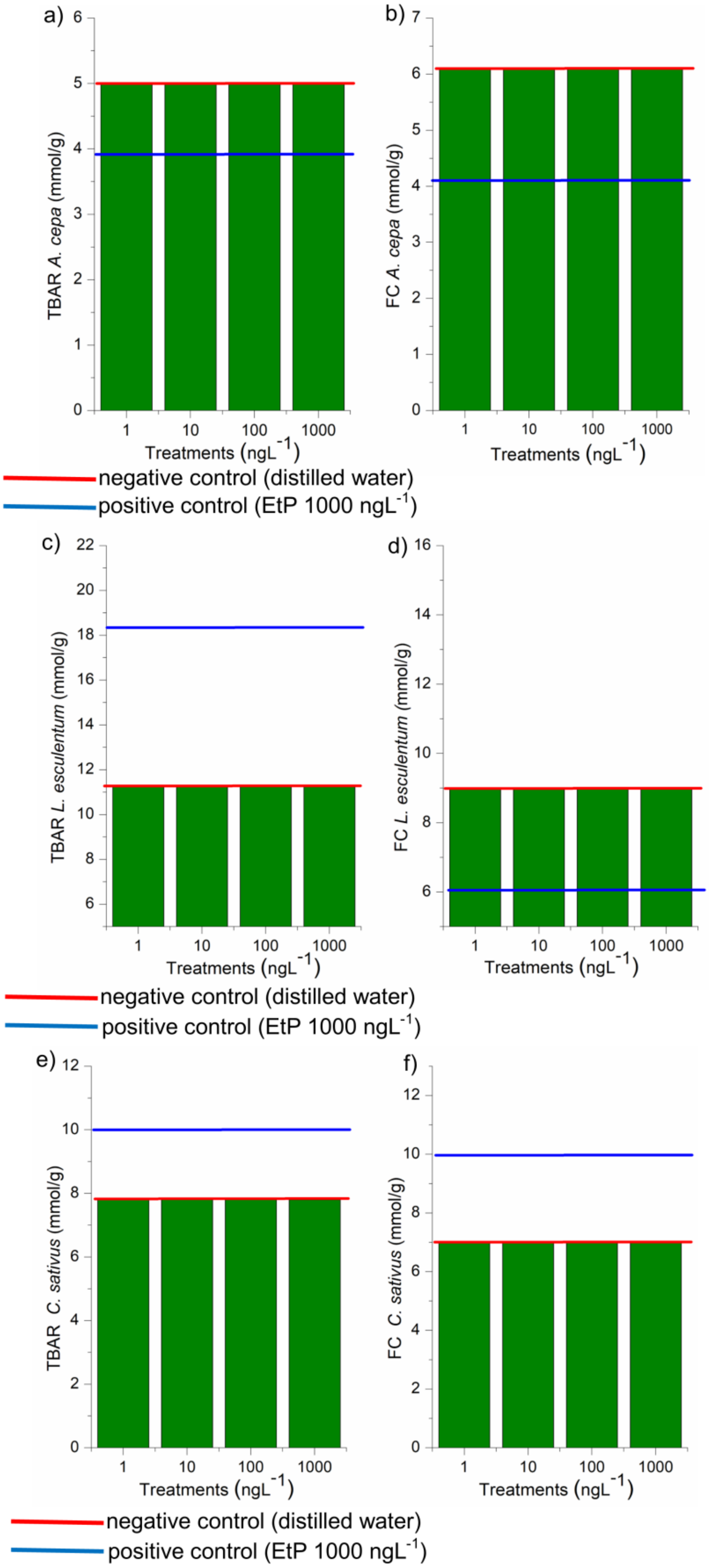

Figure S3. Lipid peroxidation (TBARs) and concentration of phenolic compounds (FC) in rootlets of *Daucus carota* L., *Lycopersicum esculentum* L., and *Cucumis sativus* L. exposed to tween 80 at concentrations of 1, 10, 100, and 1000 ng.L^{-1}.

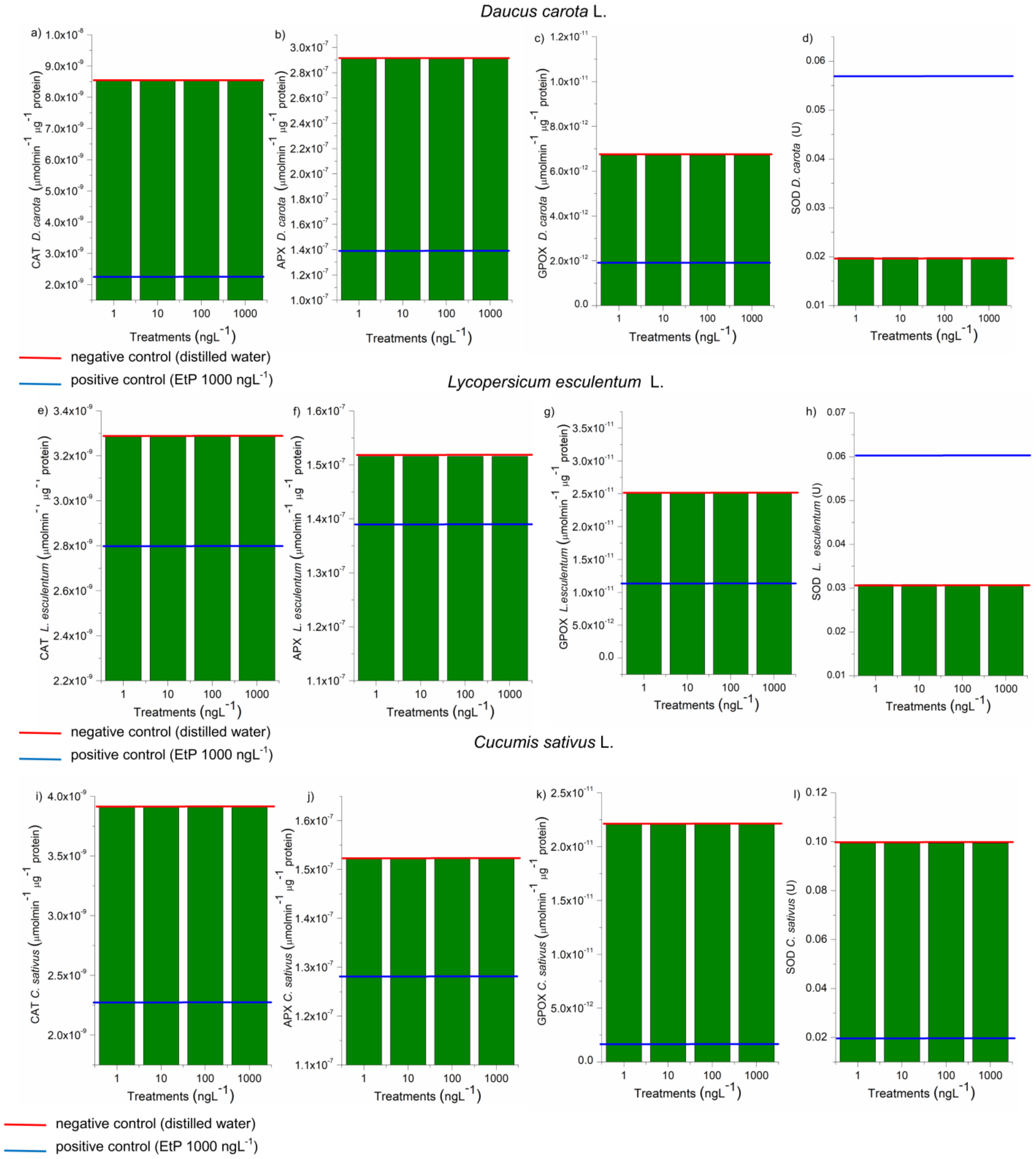

Figure S4. Modulations of the enzymes catalase (CAT), ascorbate peroxidase (APX), guaiacol peroxidase (GPOX), and superoxide dismutase (SOD) in roots of *Daucus carota* L., *Lycopersicum esculentum* L., and *Cucumis sativus* L. exposed to tween 80 in concentrations 1, 10, 100, e 1000 ng.L^{-1} .

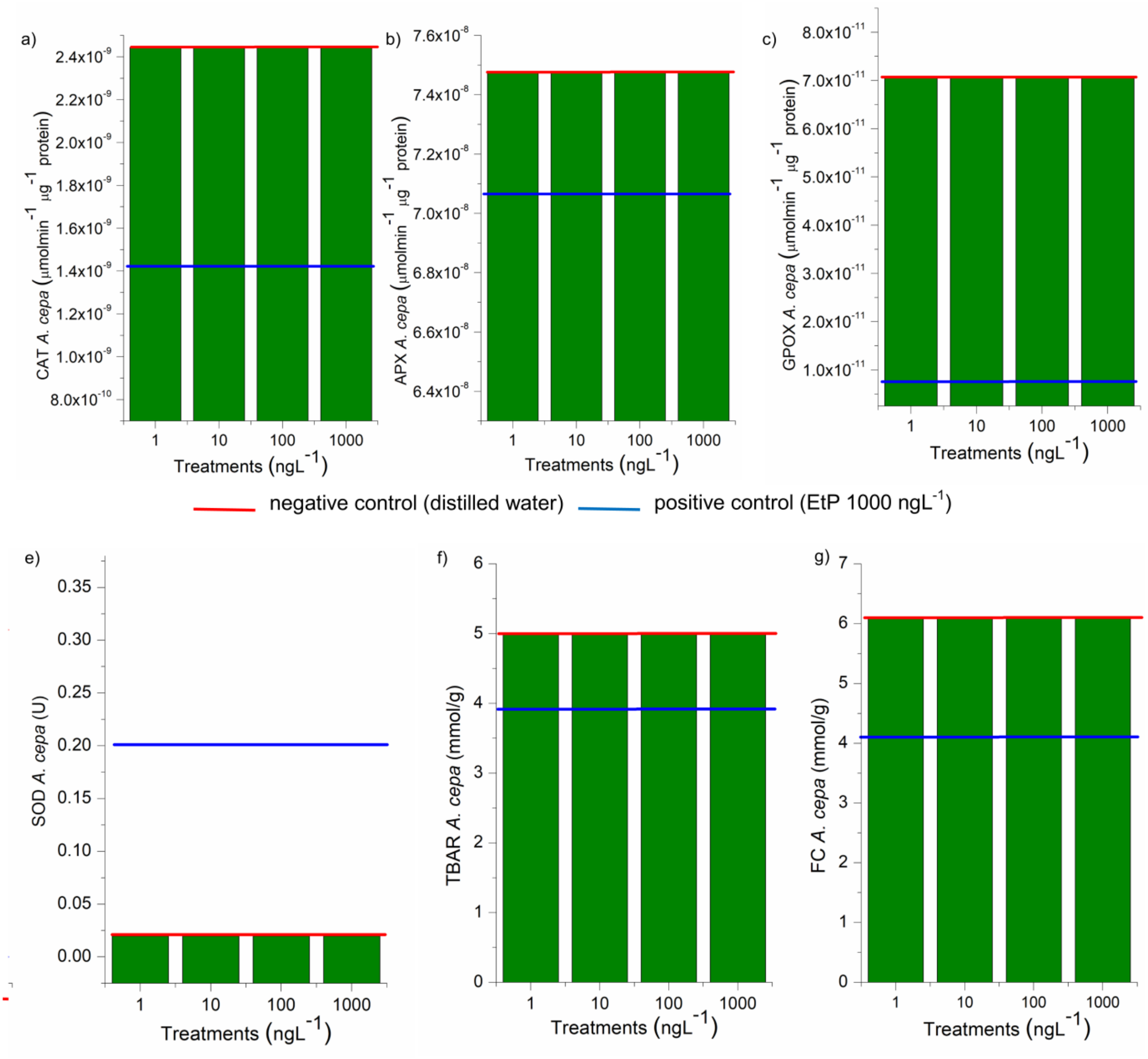

Figure S5. Modulations of the enzymes catalase (CAT), ascorbate peroxidase (APX), guaiacol peroxidase (GPOX), and superoxide dismutase (SOD) in roots of *Daucus carota* L., *Lycopersicum esculentum* L., and *Cucumis sativus* L. exposed to tween 80 in concentrations 1, 10, 100, e 1000 ng.L^{-1}.
